# Metallic bionanocatalysts: potential applications as green catalysts and energy materials

**DOI:** 10.1111/1751-7915.12801

**Published:** 2017-08-22

**Authors:** Lynne E. Macaskie, Iryna P. Mikheenko, Jacob B. Omajai, Alan J. Stephen, Joseph Wood

**Affiliations:** ^1^ School of Biosciences University of Birmingham Edgbaston Birmingham B15 2TT UK; ^2^ School of Chemical Engineering University of Birmingham Edgbaston Birmingham B15 2TT UK; ^3^ Present address: Department of Biological Sciences Faculty of Sciences, Thompson Rivers University 805 TRU Way V2C 0C8 Kamloops, British Columbia Canada

## Abstract

Microbially generated or supported nanocatalysts have potential applications in green chemistry and environmental application. However, precious (and base) metals biorefined from wastes may be useful for making cheap, low‐grade catalysts for clean energy production. The concept of bionanomaterials for energy applications is reviewed with respect to potential fuel cell applications, bio‐catalytic upgrading of oils and manufacturing ‘drop‐in fuel’ precursors. Cheap, effective biomaterials would facilitate progress towards dual development goals of sustainable consumption and production patterns and help to ensure access to affordable, reliable, sustainable and modern energy.

## Introduction

In the late 1990s, bacteria were reported to recover soluble palladium (II) via reduction into cell‐bound precious metal (PM) nanoparticles (NPs) (Lloyd *et al*., 1998) with high catalytic activity (Baxter‐Plant *et al*., 2003). Many authors have reiterated the scope for bio‐PM catalysts (e.g. reviews by Deplanche *et al*., 2011; De Corte *et al*., 2012; Castro *et al*., 2014; Kulkarni and Maddapur, 2014; Rai *et al*., 2015; Singh, 2015; Ashok, 2016). A consultancy report (Catalytic Technology Management, unpublished, 2009) concluded that a ‘me too’ catalyst must be more active than those currently available, cheaper or both. The paradigm bacterial ‘bio‐Pd(0)’ has significant potential applications in ‘green chemistry’ and environmental nanotechnology, but the criteria cannot yet be met in full due to high costs of (i) growing dedicated bacteria and (ii) precious metals; (iii) retention of the catalyst for re‐use and (iv) potential catalyst poisoning at high reaction temperatures (e.g. by sulfur via degradation of the biomaterial). Waste yeast and bacteria have been successfully reused in ‘second life’ following primary fermentations (Dimitriadis *et al*., 2007; Orozco *et al*., 2010; Zhu *et al*., 2016) while waste precious metals have been bio‐reprocessed into active neo‐catalysts (e.g. Mabbett *et al*., 2006; Deplanche *et al*., 2007; Yong *et al*., 2010, 2015; Murray *et al*., 2017a). Metal attrition from cells was negligible, enabling catalyst re‐use [e.g. 6 cycles (Bennett *et al*., 2013)] and also as an immobilized catalyst (Beauregard *et al*., 2010). The highest ‘green’ potential probably lies in ‘tandem’ one‐pot reactions which (e.g.) combine a biotransformation with a bio‐Pd‐catalysed step [e.g. in an enantioselective deracemization reaction (Foulkes *et al*., 2011)] although the low Pd loading necessary to permit continued physiological activity may not be optimal for chemical catalysis.

Concerns about nanoparticles in the environment (Valsami‐Jones and Lynch, 2015) (biomass will eventually decompose) may restrict pollutant remediation to *ex‐situ* applications. Catalyst poisoning is less relevant in a ‘dirty’ process, but this requires cheap, disposable catalyst. A life cycle analysis is needed to determine where bio‐metallic catalysts may outcompete traditional comparators, taking into account socio‐environmental as well as economic factors.

Bio‐precious metal materials are emerging in energy applications (Table 1). Large‐scale oil production may justify a once‐through catalyst if a low‐grade mixed metal ‘dirty’ catalyst can be used. On the other hand, in synthesis of fuel precursors from waste CO_2,_ some bio‐nanoparticles [e.g. structured Pd/Au core shells (Deplanche *et al*., 2012)] could potentially be used for making (e.g.) formic acid electrochemically (Humphrey *et al*., 2016). Liu *et al*. (2016) reported a bio‐Pd/Au alloy with electrocatalytic activity, but the thickness of the Pd‐shell is critical for product selectivity [e.g. for formate production a Pd‐shell of 10 nm is optimal (Humphrey *et al*., 2016)]; such fine structure control is probably beyond the reach of biosynthetic capability. Moreover, electrocatalysis does not generate longer chain hydrocarbons and this method may not be readily scalable.

**Table 1:** Microbial precious metal nanoparticles and catalysts in energy applications

**Test**	**Comments**	**Ref**
(a) Fuel cells (anodic and cathodic FC electrocatalysts)		
Anode (PEMFC)	Bio‐PM catalyst on *Desulfovibrio desulfuricans*. Required sintering to carbonize Power outputs: commercial Pt‐ FC, 200 mW; commercial Pt FC catalyst 170 mW; Bio‐Pt, 170 mW; bio‐Pd, 140 mW*. Metal content was 20 wt%; activated carbon was 80 wt% plus residual biomass component. Loading: 1 mg metal cm^−2^. *Power density was 9 mW cm^−2^ (electrode area 16 cm^2^: ref 3; c.f. ref 6).	1
Anode (alkaline FC)	Bio‐Pt catalyst made from waste yeast cells from fermentation, immobilized in polyvinyl alcohol. Activity was ~half that of commercial Pt on carbon catalyst Loading 10 mg Pt cm^−2^	2
Anode (PEMFC)	Anode as in 1. Power outputs from bio‐Pd were: *D. desulfuricans*, 142 mW; *Cupriavidis metallidurans* CH34, 68 mW; *Escherichia coli* MC4100, 29 mW, *E. coli* IC007, 115 mW; *E. coli* IC007 (made from industrial PM waste), 68 mW. Increasing Pd loading onto cells from 5 wt% to 25 wt% doubled power output	3
Anode (PEMFC)	Anode as in 1. Power outputs from bio‐Pd were: *Rhodobacter sphaeroides* 001 (biohydrogen‐producing), 20 mW and *E. coli* (‘second life’ cells from biohydrogen process): HD701, 28 mW; MC4100, 18 mW; IC007, 56 mW.	4
Anode (PEMFC)	Bio‐Pd on *Shewanella oneidensis* MR‐1. Formate was e‐donor in Pd‐NP synthesis (NP size 50‐10 nm). Pd loading was 20 wt% on cells and 1.28 mg Pd cm^−2^ on anode. Power density was 4.8 mW cm^−2^ for bio‐Pd and 5.3 mW cm^−2^ for commercial Pd‐catalyst.	5
Catalyst mix pd/activated carbon in EPR	EPR showed more electronic interactions between bio‐Pd/C than commercial Pd/C; quenching of free radicals (FR) of activated carbon was higher with sintered bio‐Pd_*D. desulfuricans*_ than with bio‐Pd_*E. coli*_; both bio‐Pds gave higher FR quenching than Pd/C catalyst.	6
Native cells in rotating disc electrode	Pd loading 20 wt% (*E. coli*). Use of formate as *e* ^−^ donor for Pd(0) formation gave small, well separated NPs with no electrochemical activity. Bio‐Pd made under H_2_ showed proton adsorption/desorption. Similar results obtained using bio‐Pd on *Shewanella oneidensis*.	7
Cyclic Votammetry	Palladium NPs on *D. desulfuricans* (native cells). Pd loading not stated Proton‐concentration gradients involved in extracellular electron transfer processes. Pd‐NPs proposed to augment natural *e* ^−^ transport chain. Activity increased by adding formate (live cells only).	8
Cathode (PEMFC)	Material as in 1. Bio‐Pd_*E.coli*_ at 25 wt% Pd. Commercial anodic catalyst in FC test rig Paxitech FCT‐50s. Cathodic activity of bio‐Pd was 25% of that of commercial catalyst. Combining with bio‐Pd with Pt (various ratios) did not significantly increase power output	9
O‐reduction reaction: cyclic voltammetry	Bio‐Pt_*E. coli*_ cleaned in NaOH gave enhanced cyclic voltammetry response, cf. sintered material. Bio‐Pt_*D. desulfuricans*_ was better when cleaned in phenol–chloroform. Max. current was 8.5 μA and 25 μA, respectively (glassy carbon rotating disc electrode) (see Table 2)	10

Application	Comments	Ref
(b) Upgrading of 5‐hydroxymethyl furfural (5‐HMF) into 2,5, dimethyl furan (drop‐in fuel)		
DMF production	Pd‐based catalyst supported on *Bacillus benzeovorans* (Table 3). The conversion was the same as Pd/C catalyst, but selectivity was higher	11
(c) Catalytic upgrading of pyrolysis oil		
Pyrolysis oil algal (*Chlorella*)	Oil was from algae (*Chlorella*) slurry. Bio‐Pd_*D. desulfuricans*_ (5 wt% Pd:biomass; 5 wt% catalyst per reaction) and Pd/C (10% wt% catalyst per reaction: 15 g oil; 4 h; 325°C) had similar activities, O_2_ and N_2_ contents were reduced by 65% and 35% respectively. Bacterial component of catalyst contributed to bio‐oil yield. Nanoparticle size increased in bio‐catalyst but not Pd/C. Catalysts were reclaimed but not evaluated in repeated reactions.	12
Pyrolysis oil beechwood	Oil was from beechwood (fast‐pyrolized 500°C; < 2 h). Catalysts: 5 wt% Pd_*E. coli*_, 5 wt% Pd/C and commercial Pd/Al_2_O_3_ (0.6 g catalyst to 20 g oil). Minimum viscosity was ~ 0.035 Pas (250°C, 3 h), increasing thereafter. Bio‐Pd gave similar deoxygenation (~20%) at 160°C but performed less well at higher temperatures, attributed to higher coking than with Pd/C at > 259°C (see Fig. S1, Table S1)	13
Pyrolysis oil pine wood	Made from beetle‐killed pine trees which were more easily pyrolysed; not tested for upgrading via bio‐derived catalysts	14
(d) Catalytic upgrading of heavy oils		
Upgrading of heavy fossil oils from Touchstone (Canada)	Production of bio‐magnetite (Fe_3_O_4_) by *Geobacter sulfurreducens* that reduces Fe(III) to Fe(II) thence to bio‐magnetite. Functionalization of bio‐magnetite with Pd(0) onto bio‐magnetite. Comparable oil upgrading to commercial alumina‐supported catalysts. High liquid yield (90%); less viscous than without catalyst. Coking reduced twofold‐ to fourfold using 9.5 wt% Pd. In situ process proposed by stimulation of natural geomicrobiological system	15
	Bio‐PMs made on cells of *Bacillus benzeovorans* and *Desulfovibrio desulfuricans*. Bio‐ Pd/Pt mix (1:1) was better than Pd alone. Comparable upgrading to commercial catalyst via using 5 wt% and 20 wt% metal loading. Liquid yield was 90%. Coking was reduced by ~20% as compared to using commercial catalyst (Ni‐Mo/Al_2_O_3_). Extensive catalyst characterization.	16
	Catalyst (Bio‐Pd/Pt; 2.5 wt% Pd/2.5 wt% Pt) was made on *B. benzeovorans* and *E. coli*. The former showed ~30% reduced coking. It was discussed that the catalyst may reduce V(V) to a V‐species that does not promote free radical reactions leading to coking. The higher inorganic phosphate content of the Gram‐positive cell wall (teichoic acids) may sorb Ni^2+^ (although the cells become carbonized to become part of the fuel). Similar results were obtained using metals sourced from simulated road dust waste leaches	17
(e) Other opportunities		
Dry reforming of methane (DRF)	DRF makes syngas from CH_4_ + CO_2_ over (e.g.) Ni‐catalyst. Catalyst deactivates due to carbon deposition; noble metals (alone or with Ni) are longer lived. Bio‐PM catalysts have not been evaluated but show coking resistance (above)	18
Upgrade waste glycerol	Bio‐Au(0) nanocatalyst from jewellery waste was shown to oxidize glycerol to glycerate Application to biodiesel waste glycerol is not yet shown. Glycerate is a substrate for bio‐H_2_ production	19
Photocatalytic water splitting to make H_2_	Noble metals are used to split water to make H_2_. (Bio‐PMs are not yet tested)	20
	Optically active ZnS was made using mine drainage treatment bioprocess off‐ gas (H_2_S)	21
	CuS has been separated from other metals in biogenic metal recovery from waste	22
	CuS/ZnS hybrid (chemically made) was used in photocatalytic water splitting	23
	CdS quantum dots were made with bacteria and cysteine (Cd is more toxic than Zn; an energy application was not reported).	24
	Biogenic metal selenides were made *ex‐situ* (reduction of selenite by *Veilonella atypica)*	25
	CdSe can be made by *Fusarium oxysporum* (intracellularly; metabolic process)	26
	CdSe‐hydrogenase hybrids/artificial hydrogenases are reported for water splitting	20

1. Yong *et al*. (2007). 2. Dimitriadis *et al*. (2007). 3. Yong *et al*. (2010). 4. Orozco *et al*. (2010). 5. Ogi *et al*. (2011). 6. Carvalho *et al*. (2009). 7. Courtney *et al*. (2016). 8. Wu *et al*. (2011). 9. Stephen, A.J. unpublished data. 10. Williams (2015). 11. Omajali (2015). 12. Kunwar *et al*. (2017). 13. Deilami *et al*. (unpublished). 14. Luo *et al*. (2017) 15. Brown *et al*. (2016). 16. Omajali *et al*. (2017). 17. Murray *et al*. (2015). 18. Pakhare and Spivey (2014). 19. Deplanche *et al*. (2007). 20. Ran *et al*. (2014). 21. Murray *et al*. (2017b). 22. Nancucheo and Johnson (2012). 23. Zhang *et al*. (2011). 24. Yang *et al*. (2015);. 25. Fellowes *et al*. (2013). 26. Yamaguchi *et al*. (2016).

As an alternative, chemical upgrading of CO_2_ into hydrocarbon fuels is scalable. Recent advances employ the reverse water gas shift reaction (reduction of CO_2_ to CO) in tandem with Fischer–Tropsch chemistry to convert the more reactive CO into hydrocarbons. Consuming CO (a catalyst poison) shifts the equilibrium towards products in the reverse water gas shift reaction, promoting CO_2_ consumption. An efficient, abundant, low‐cost catalyst(s) must give product selectivity in the desired range (~C5–8 for gasoline; *˜*C9–16 for diesel fuel). Precious metals (Pd, Pt, Ru, Rh) on SiO_2_ have been used in the reverse water gas shift reaction, while catalysts for the Fischer–Tropsch process are usually Fe or Co‐based, with recent innovations towards one‐pot reactions (see e.g. Mattia *et al*., 2015; Owen *et al*., 2016; Prieto, 2017) following early work (Dorner *et al*., 2010) that showed conversion of CO_2_ to hydrocarbons (41% conversion and a C2–C5+ selectivity of 62%) using a doped Fe‐based system. The potential for using biogenic catalysts has not been explored although a paradigm hybrid bio‐magnetite/Pd(0) catalyst has been reported (Brown *et al*., 2016). When made from waste, biogenic precious metal catalysts (e.g. Pd/Pt mixtures ~30% Pd) also contained other metals, for example from Degussa processing waste: Al (42%), Ag (6%) and Mg (3%) and from spent automotive catalyst leachate: Fe (14%), Mg (12%) and Al, 27%), i.e. metals that were present in the original solid material (Mabbett *et al*., 2006; Macaskie *et al*., 2010). Potentiation of catalytic activity occurred (e.g. more than 10‐fold in the case of reduction of Cr(VI) to Cr(III)) using these waste‐derived mixed metal catalysts (Macaskie *et al*., 2010). Hence, exploration of bio‐catalyst made from automotive leachate may be warranted for CO_2_ valorization, particularly as waste bacteria for use as catalyst support (Orozco *et al*., 2010; Zhu *et al*., 2016; Stephen *et al*., this volume) are readily available from other scalable biotechnology processes. However, selectivity towards specific hydrocarbon products may not be compatible with such economies.

In contrast to CO_2_‐valorization, ‘proof‐of‐principle’ application of biogenic catalysts has been shown in four key areas of energy and fuels (Table 1).

## Fuel cell electrocatalysts

Fuel cells comprise an anode [where fuel, e.g. H_2,_ is split catalytically to give electrons (current) and protons], a cathode (where protons combine with O_2_ in air to give water) (Kraytsberg and Ein‐Eli, 2014) and an electrolyte that allows passage of positive ions between them (Kirubakaran *et al*., 2009). The polymer electrolyte fuel cell (PEM fuel cell) uses purified hydrogen at low temperatures (80°C) with rapid start up (Mehta and Cooper, 2003). PEM fuel cells are applicable for use in (e.g.) vehicles (Hannan *et al*., 2014) or larger ‘stacks’ for domestic power (Staffell *et al*., 2015). Durability targets (internationally) are 5000 h and 40 000 h of operation for automotive and stationary fuel cells, respectively (Rice *et al*., 2015). The US Department of Defence installed 5 kW PEM fuel cell systems in ~40 military bases (cost of > $100 000 per system) but, with an operational lifespan of only 500 h, the systems required overhauling annually (Staffell *et al*., 2015). The cost of precious metal catalysts is restrictive; other catalysts are under development but the power to weight ratio is key, especially for portable and aerospace applications. Substitution of fuel cell Pt‐electrocatalysts (0.2–0.8 mg cm^−2^) would use lighter, equivalently performing (but robust against the high local acidity) metals like Pd (e.g. Meng *et al*., 2015; Gómez *et al*., 2016), particularly in the cathodic reduction of O_2_ (He *et al*., 2005) which is rate‐limiting. Reducing Pt costs/loadings could also be achieved by optimizing Pt nanoparticles (via size control and increased uniformity), or by developing alloys (Zhu *et al*., 2015). Developments towards microbially derived fuel cell catalysts are shown in Table 1. The anodic reaction is well reported, and the challenge is now to develop an efficient cathodic oxygen reduction catalyst; electrochemical test data are shown in Table 2.

**Table 2:** Activity of bio‐Pt catalyst on *Escherichia coli* and *Desulfovibrio desulfuricans* compared to commercial TKK fuel cell catalyst

Material/treatment	Specific activity (mA cm^−2^)	Mass activity (mA mg Pt^−1^)	No. electrons transferred per O_2_
Bio‐Pt_*E. coli*_ (NaOH)	0.68 ± 0.15	75 ± 17	3.78 ± 0.23
Bio‐Pt_*D. desulfuricans*_ (phenol–chloroform)	1.43 ± 0.28	304 ± 53	3.84 ± 0.12
TKK catalyst	0.45 ± 0.02	374 ± 4	3.86 ± 0.07

Taken from Williams (2015). Pt loading was 5 wt% of the biomass. Bio‐Pt_*E. coli*_ was cleaned using NaOH. Bio‐Pt _*D. desulfuricans*_ was cleaned using phenol–chloroform. As with the anodic and cathodic tests in the PEM fuel cell (Table 1), the *E. coli* biomaterial was ~25% as active (mass activity) as that from *D. desulfuricans* and had ~ half the specific activity (mA cm^−2^). However, growth of *E. coli* is readily scalable and it makes active bio‐metal catalyst when used in ‘second life’ following an independent primary fermentation (Orozco *et al*., 2010; Zhu *et al*., 2016). In contrast to *E. coli*,* D. desulfuricans* cells are obligately anaerobic, growth is less readily scalable, and they produce H_2_S, a powerful catalyst poison that requires more extensive washing of the cells prior to use. However, a metal bioremediation process that couples excess biogenic H_2_S (used for minewater clean‐up with respect to heavy metals (Hedrich and Johnson, 2014)) also produces waste biomass of a sulfate‐reducing bacterial consortium which may find a ‘second life’ use as a bio‐metallic catalyst for fuel cell application, mitigating waste disposal costs.

## 2,5‐dimethyl furan (DMF) production from 5‐hydroxymethyl furfural (5‐HMF)

Carbohydrates form ~75% of the annual renewable biomass (Schmidt and Dauenhauer, 2007). In thermochemical hydrolysis [e.g. of fermentation feedstocks: Orozco 2011], 5‐hydroxymethyl furfural is produced via breakdown of hexoses in cellulose and starch hydrolysates (Román‐Leshkov *et al*., 2007; van Putten *et al*., 2013). 5‐hydroxymethyl furfural is a precursor to 2, 5‐dimethylfuran (DMF), a ‘drop‐in’ fuel for conventional engines (Zhorg *et al*., 2010). DMF contains comparable energy to gasoline (Davis *et al*., 2011) (energy contents are 31.5 MJ l^−1^ and 35 MJ l^−1^ respectively). DMF is also more advantageous than ethanol because of its higher gravimetric energy density (about 40%), higher boiling point and insolubility in water; hence, it is a potential alternative biofuel (Tian *et al*., 2011).

Various catalytic applications have been developed to achieve good yield and selectivity to 2, 5‐dimethyl furan. Most have focused on commercial heterogeneous mono and bimetallic catalysts based on (e.g.) Ru, Pd, Pt, Au, Cu (Tong *et al*., 2011; Hansen *et al*., 2012; Nishimura *et al*., 2014; Zu *et al*., 2014; Luo *et al*., 2015) which are costly. A bacterial platform would provide a cheaper, sustainable source of supported precious metal catalyst, possibly using metals biorefined from waste sources. As the first step, conversion of 5‐HMF to DMF using a bio‐Pd‐based catalyst and a Pd/carbon catalyst was compared (Omajali, 2015), with better selectivity to DMF observed using the biomaterial (Table 3). Nishimura *et al*. (2014) showed application of a Pd/Au bimetallic/C catalyst and hence the use of bio‐Pd/Au (Deplanche *et al*., 2012; Hosseinkhani *et al*., 2012; Liu *et al*., 2016) is worth evaluating in this application.

**Table 3:** Pd‐catalyst‐mediated upgrading of 5‐hydroxymethyl furfural into 2,5‐dimethyl furan

Catalyst and H‐donor	5‐HMF conversion (%)	DMF yield (%)
5 wt% Pd/carbon (formic acid)	97.5	26.5 ± 2.0
Bio‐Pd‐based (5 wt% metal; formic acid)	96.8	49.8 ± 0.6
5 wt% Pd/carbon (2‐propanol)	94.5	32.6 ± 1.8
Bio‐Pd‐based (5 wt% metal; 2‐propanol)	94.5	42.6 ± 1.2

Taken from Omajali (2015). Bio‐catalyst was prepared on cells of *Bacillus benzeovorans*. *Bacillus* was selected because this genus is grown at large scale for commercial production of enzymes; a cost‐benefit analysis for ‘second life’ production of catalyst as compared to other current routes for disposal of waste biomass is required.

## Upgrading of pyrolysis oils

Hydrothermal liquefaction (HTL) and fast pyrolysis are thermal treatments of wet organic feedstocks (e.g. agri‐food wastes, manure, algae) which produce a highly viscous biofuel (pyrolysis oil). This is unsuitable as an alternative to fossil fuels without further processing. In addition to high viscosity, crude bio‐oil contains large quantities of oxygenated molecules which are unsuitable for use directly in vehicle engines and are incompatible for blending with fossil fuels, without further upgrading to remove oxygen (hydrodeoxygenation, HDO).

Hydrodeoxygenation is well studied, but a mechanistic understanding for disassembly of biopolymers and their subsequent deoxygenation is incomplete. New catalytic routes are required to make cost effective drop‐in fuels (Huber *et al*., 2006; Rinaldi and Schuth, 2009). Precious metal‐based catalysts feature prominently (e.g. Bouxin *et al*., 2017), and theoretical chemistry can play an important role in understanding the competing reactions on the surface of, for example, Pt (111) faces (Liu *et al*., 2017). However commercial precious metal catalysts would be uneconomic; moreover, fuel cell electrocatalysts (above) will compete in parallel for limited global resources. The energy demand of winning precious metals from primary ores (e.g. 14 t of CO_2_ is emitted per kg of Pt produced: Anon 2008) is a major consideration; carbon‐neutral fuel needs, in itself, to produce a low carbon footprint.

Catalytic deoxygenation reactions include dehydration, hydrogenolysis, hydrogenation, decarbonylation and decarboxylation (Fig. S1, Table S1). For fuels in the diesel range, C‐C coupling reactions can be achieved through routes such as aldol‐condensation, ketonization, oligomerization and hydroxyalkylation, but hydrodeoxygenation is currently considered the most effective method for bio‐oil upgrading, improving the effective H/C ratio and leading to hydrocarbons.

To date, several classes of catalysts are reported for hydrodeoxygenation. In addition to precious metal catalysts, other metals (Fe, Ni and Cu) have shown good selectivities in hydrogenation and hydrogenolysis reactions. However, high hydrogen pressures can lead to complete hydrogenation of double bonds (Bykova *et al*., 2012). Industrial catalysts based on Co‐Mo and Ni can provide good hydrodeoxygenation performance, but these deactivate rapidly due to coke formation and water poisoning (Badawi *et al*., 2011).

The acidic, corrosive biofuels also limit catalyst lifetime and compromise the process economics. Work has focused on upgrading of biocrude oils using various catalysts (e.g. Co‐Mo, Ni‐Mo Pd/C (e.g. Biller *et al*., 2015; Si *et al*., 2017)). Of these, Pd (which is a noble metal and hence is dissolution tolerant) is promising. 5‐hydroxymethylfurfural is made within in the product oil (Dang *et al*., 2016), enabling possible catalytic conversion into 2,5‐dimethyl furan into a second fuel stream.

Despite the high costs, precious metal‐based catalysts are favoured for bio‐oil upgrading (see reviews: Watson, 2014; Pandey *et al*., 2015; Cheng *et al*., 2016; Lee *et al*., 2016; Nam *et al*., 2017; Fermoso *et al*., 2017); bio‐manufactured catalyst should now be added to the portfolio (Table 1). Bio‐reprocessed precious metal waste has not yet been tested as a cheap metal source (c.f. below). The upgrading efficiency, product spectrum and cost savings are being factored into ongoing research via life cycle analysis, towards the dual development goals of sustainable consumption and production patterns for affordable, reliable, sustainable (non‐fossil) energy.

## Upgrading of fossil oils

The hydrogen economy and carbon‐neutral biofuels lag behind the timeline for change articulated by the Stern Review (Stern, 2006). Hence, cleaner production of fossil fuels is vital as these will remain the predominant short‐term sources of supply (~80% of global needs; Anon 2014). With globally declining light crude oil reserves, heavy oil and bitumen use will increase from 2 to 7 million barrels day^−1^ by 2030 (Anon 2015). Oil sands production emits more greenhouse gas and uses more water than conventional light oil production (Findlay, 2016) and additional refining is also required (Huc, 2011). Heavy oil exploitation is complicated by the high viscosity, low hydrogen content and high amounts of resins and asphaltenes. (Bio)geochemical processes evolved the hydrocarbons, leading to materials with high contents of heavy molecules rich in sulfur, nitrogen, oxygen and metals (e.g. Ni, V), with high viscosity and acidity (Head *et al*., 2003; Huc, 2011). Technologies for heavy oil upgrading have been reviewed (Heraud *et al*., 2011; Castaňeda *et al*., 2014). Upgrading *in situ* gives a cleaner production of less viscous oil, which is more easily transported without the use of diluents (Shah *et al*., 2010). The THAI‐CAPRI (Toe‐to‐Heel Air Injection coupled with Catalytic Upgrading Process *In situ*) technology combines thermally enhanced oil recovery with down‐hole catalytic upgrading of heavy oil into light fractions (Greaves and Xia, 2004; Hart *et al*., 2014). This catalytic upgrading, using steam, hydrogen and methane, showed significant improvements over non‐catalytic thermal processes (Hart *et al*., 2014). Conventional cracking catalysts such as supported noble metals are prohibitively expensive; one economic option could utilize regenerated catalysts from treated oils but these have lower activity (Hart *et al*., 2014).

The future is uncertain. Healing (2015) questioned the economics of the THAI process, Findlay (2016) discussed this in view of wider issues (regulation, cost and price uncertainties), and Nduagu *et al*. (2017) discussed the wider issues of oil sands processes in terms of performances and economics including greenhouse gas emissions and other environmental impacts. Meanwhile, emerging researches include modelling of the THAI‐CAPRI process (Ado *et al*., 2017) and the successful use of bio‐nanoparticle catalysts sourced from wastes (Table 1). Application of nanoparticles in enhanced oil recovery has been reviewed (Negin *et al*., 2016; Sun *et al*., 2017). While bio‐nanoparticles are held immobilized on bacterial cells, thermal degradation would evolve them in association with biomass‐carbon. Environmental concerns about nanoparticles have been expressed (see earlier), but it is also argued that naturally occurring nanoparticles are ubiquitous in the environment (Montaňo *et al*., 2014) while evidence for natural biogeochemical cycling of platinum has been reported (Reith *et al*., 2016).

## Conclusions and future scope

As far as we are aware, this is the first overview of the potential for biogenic catalysts in various energy applications within the ‘whole energy mix picture’. The concept of using microbial technologies to make materials for application to sustainable energy‐generating processes (as compared to energy and waste savings via use in ‘green chemistry’) is a new direction. Palladium occurs in spent nuclear fuels (one ton of spent nuclear fuel contains > 2 kg of Pd or 10% of global requirements: Bourg and Poinssot, 2017). Even though radiation‐resistant bacteria are well known, and the ability of microorganisms to discriminate between isotopes of essential metals (Fe, Mo) has been reported (Wasylenki *et al*., 2007), separation of the active ^107^Pd (half‐life 6.5 m yrs; 15% of the Pd inventory) from the stable isotopes (85%) is probably beyond the reach of 21st‐century biotechnology. Moreover, while biorecovery of precious metals from aggressive solutions (using pre‐palladized cells, via chemical catalysis using Pd‐bio‐nanoparticle ‘seeds’) has been shown (Murray *et al*., 2017a), the metal composition of the neo‐catalyst reflects the metallic composition of the waste (Macaskie *et al*., 2010), and hence, selective biorecovery of Pd against higher active radionuclide contaminants would be prohibitively difficult.

Photochemical water splitting to make clean hydrogen is a well‐established solar technology, best achieved traditionally using noble metal catalysts (Ran *et al*., 2014). As potential alternatives, various biogenic, optically active, materials have been made (metal sulfides, selenides; Table 1) but as far as we are aware these have not yet been tested in this application. Biotechnologically, hydrogenase‐metal selenide hybrids show potential (Ran *et al*., 2014). Economic attractiveness is boosted by the potential fabrication of such materials from metallic or H_2_S wastes (Nancucheo and Johnson, 2012; Murray *et al*., 2017b) but, given that the elements are abundant and cheap, the main driver may be waste valorization and mitigation of disposal costs, while incorporation of even a small amount of metal impurity may affect the optical property, and hence, neo‐material from metallic waste may not be a useful option.

For biofuels, an unexpected ‘biotechnology’ has added value towards pine wood‐derived pyrolysis oil. Very large areas of pine forest in the USA have been killed by beetles, producing very dry, porous wood. This enables the use of larger wood chips (Luo *et al*., 2017), reducing comminution costs and energy use. It would be interesting to apply novel biogenic catalysts to pyrolysis oil obtained from this material.

## Conflict of interest

None declared.

## Supporting information


**Fig. S1.** Major reactions occurring during bio‐oil hydrodeoxygenation.
**Table S1.** Comparison of 5 wt% Pd on carbon catalyst and 5 wt% bio‐Pd.
