# Supplementary material for: Metallic bionanocatalysts: potential applications as green catalysts and energy materials
Source: Microb Biotechnol. 2017 Aug 22;10(5):1171–80. doi: 10.1111/1751-7915.12801 (PMC5609244; doi:10.1111/1751-7915.12801)
Supplement: Supplementary file 1 — Fig. S1. Major reactions occurring during bio‐oil hydrodeoxygenation. Table S1. Comparison of 5 wt% Pd on carbon catalyst and 5 wt% bio‐Pd. [file MBT2-10-1171-s001.doc]

**Fig. S1:** Major reactions occurring during bio-oil hydrodeoxygenationtions occur


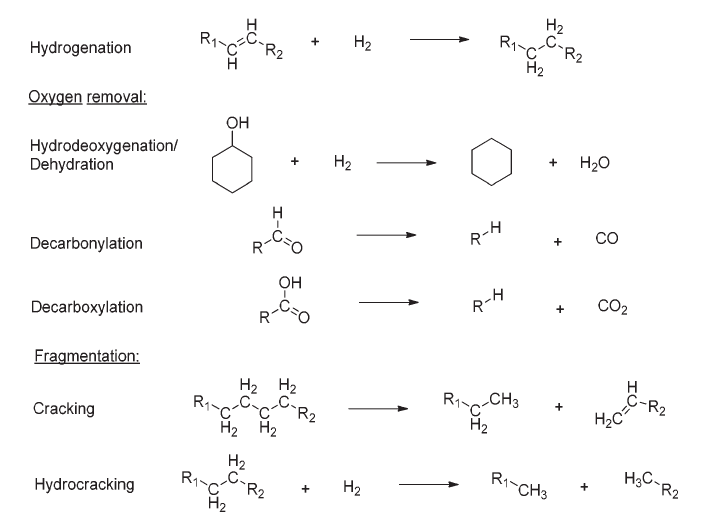


Taken from Zacher, A.H., Olarte, M.V., Santosa, D.M., Elliott, D.C. and Jones S.B. (2014) A review and perspective of recent bio-oil hydrotreating research.  *Green Chem.* **16**: 491-515.

**Table S1**: Comparison of 5 wt% Pd on carbon catalyst and 5 wt% bio-Pd

Pd/C catalyst Bio-Pd catalyst Crude oil

Crude oil % protons assigned by ^1^H NMR

HC=C-, phenolic H, OH 0.9 0.6 5.2

Aromatic H 1.7 2.2 0.2

Analysis by GC-MS (mol%)

Esters, ketones, alcohols 4.7 3.4 38.0

N- and O heterocyclic compounds 0 2.7 19.8

Taken from: Wood, J., Deilami, S., Kunwar, B., Macaskie, L.E. and Sharma, B.K. (2016) Catalytic Upgrading of HTL Bio-Oil Using Bio-Pd/C Catalyst.  Oral presentation at the Euro Biomass International Conference Birmingham UK, August 8-9th 2016.
